# Perceived Level of Threat and Cooperation

**DOI:** 10.3389/fpsyg.2021.704338

**Published:** 2021-07-05

**Authors:** Ori Weisel, Ro'i Zultan

**Affiliations:** ^1^Coller Scool of Management, Tel Aviv University, Tel Aviv, Israel; ^2^Department of Economics, Ben-Gurion University of the Negev, Beer Sheva, Israel

**Keywords:** intergroup conflict, cooperation, threat, framing, common enemy, social support, conservation of resources

## 1. Introduction

The evolution of parochial altruism goes hand in hand with intergroup conflict. Helping other group members is evolutionary stable in the presence of an outside threat (Bowles et al., 2003; Guzmán et al., 2007), and hostility toward other groups can evolve together with parochial altruism (Choi and Bowles, 2007; Bowles, 2008). Furthermore, group reputation fosters cooperation with fellow group members in times of conflict—even in an environment that does not foster cooperation in times of peace (Hugh-Jones and Zultan, 2013).

Social scientists have long documented that intergroup conflict increases intragroup cooperation (Sumner, 1906; Williams, 1947; Simmel, 1955; Coser, 1956). Over a century ago, Sumner (1906) wrote that “the exigencies of war with outsiders are what makes peace inside.” Indeed, prosocial behaviors, such as volunteering and blood donations, increase during times of war or exposure to terror attacks (Schmiedeberg, 1942; Janis, 1951; Glynn et al., 2003; Penner et al., 2005; Steinberg and Rooney, 2005; Gneezy and Fessler, 2012; Berrebi and Yonah, 2016). This phenomenon can be reproduced in experimental settings, either under experimentally induced external threat to the group (Wright, 1943; Feshbach and Singer, 1957; Sherif, 1961, 1966; Burnstein and McRae, 1962; Hargreaves-Heap and Varoufakis, 2002), or in times of interstate conflict (Gneezy and Fessler, 2012). Democratic leaders are also apparently aware of this phenomenon, often dubbed the *common enemy* effect, as they are more likely to initiate interstate conflict at times of internal unrest or threatened leadership (Sirin, 2011).

In this paper, we argue that outside threat has the capacity to both increase *and* decrease intragroup cooperation. We propose that the crucial psychological variable that determines the response to outside threat is the level at which threat is perceived and construed. Outside threat increases cooperation only if it menaces the group as a whole (Williams, 1947). As intergroup conflict poses a threat both to the group as a whole and to individual group members, the same threat can trigger different—and even opposing—responses, depending on how it is perceived. These perceptions are sensitive to the duration and intensity of the conflict, media coverage, and the salience of various aspects of intergroup conflict.

This *Perceived Target of Threat* principle can be summed thus: individuals who perceive the *group* to be under threat help *the group*, whereas an individual who perceives *himself* to be under threat helps *himself* (Weisel and Zultan, 2016). In the following, we review empirical support for this principle and discuss its psychological antecedents.

## 2. Mobilization in Intergroup Conflict

The most direct effect of outside threat on intra-group cooperation is on mobilization in the context of the intergroup conflict. The perception of the group being attacked leads to the well-known “rally around the flag” effect, namely an increase in support for political incumbents and military action (Mueller, 1970, 1973; Brody, 1991; Baker and Oneal, 2001; Newman and Forcehimes, 2010).

Paradoxically, however, while attacks on the group generate support for military action, with time, ingroup casualties lead people to withdraw support (Mueller, 1973; Gartner and Segura, 1998; Kuijpers, 2019). This effect is often interpreted as reflecting a rational cost-benefit calculation, with ingroup casualties seen as costs of war (Larson, 1996; Gartner, 2008a; Gelpi et al., 2009). In this view, casualties oppose, rather than moderate, the “rally around the flag” effect.

Public opinion, however, mainly reacts to local—rather than national—casualties (Gartner et al., 1997; Kriner and Shen, 2012, 2014). Furthermore, the effect of casualties decays rapidly, and is strongest in individuals who are less attentive to national and local news—where a possible explanation is that such individuals are more likely to experience the casualties information in a more personal way (Althaus et al., 2012). Most illustrative is the finding that while the events of 9/11 lead to public support for president Bush and enabled the war in Iraq, personal ties to 9/11 casualties were negatively correlated with presidential support (Gartner, 2008b). Thus—notwithstanding the acknowledged merit of the cost-benefit view—the reaction to casualties is more visceral than a cold weighing of costs.

The perceived target of threat principle provides a unifying explanation for the apparent contradiction between the “rally around the flag” and the casualties effect. We suggest that causalities shift perceptions from the group-level to the individual-level threat, thus fostering opposition to the conflict. Put in context, if an individual perceives her group to be under attack, as did most Americans during the 9/11 attacks, she will display parochial altruism and be mobilized to participate in and support the conflict effort. On the other hand, a more personal perception of the attack as threatening oneself—which evolves over time and with personal experience of casualties—is associated with a feeling of insecurity and leads the individual to withdraw support from group efforts (Gartner, 2008a,b).

## 3. Natural Threats

Some evolutionary arguments for cooperation in conflict do not extend to natural disasters (Hugh-Jones and Zultan, 2013; De Jaegher and Hoyer, 2016). When Barclay and Benard (2020) introduced external threat to a public goods game, cooperation was higher when the threat was framed as social (vs. asocial). Nonetheless, empirical studies suggest that—similar to intergroup conflict—natural disasters increase shared group identity, trust, and willingness to help (Pena et al., 2014; Vezzali et al., 2015; Drury et al., 2016; Maki et al., 2019; Lee, 2020, 2021). Post-disaster social reactions typically follow two distinct paths. Initial responses to disaster are characterized by mobilization of social support as communities cooperate in mutual help and protection. With time, social responses shift to disillusionment, and social support deteriorates (Kaniasty and Norris, 2004; Kaniasty, 2020).

We suggest that these dynamics can be viewed to go hand in hand with a shift in the perceived level of threat. A number of observations are in line with this interpretation. Tilcsik and Marquis (2013) found that, whereas small-scale disasters have a positive effect on local firms' philanthropic spending, major disasters have a negative effect. Vardy and Atkinson (2019) found, using laboratory dictator games, that exposure to people harmed by a cyclone increased prosociality, while incurring personal property damage had a negative effect on prosociality. These effects are consistent with the view that proximity and scale increase the likelihood of being affected personally by the disaster and shifting the perception of threat to the individual level, thereby reducing social cohesiveness in the community.

## 4. Experimental Team Games

Field research on mobilization in conflict and on responses to natural threats provides suggestive evidence in support of the principle of perceived level of threat. The observed behavioral patterns, however, are correlational in nature and open to different interpretations and multiple explanations. In order to isolate the perceived level of threat, we conducted laboratory experiments using team games, where the perceived level of threat can be directly manipulated in order to establish its causal effect (Weisel and Zultan,, in press; Weisel and Zultan, 2016).

**Figure 1:**
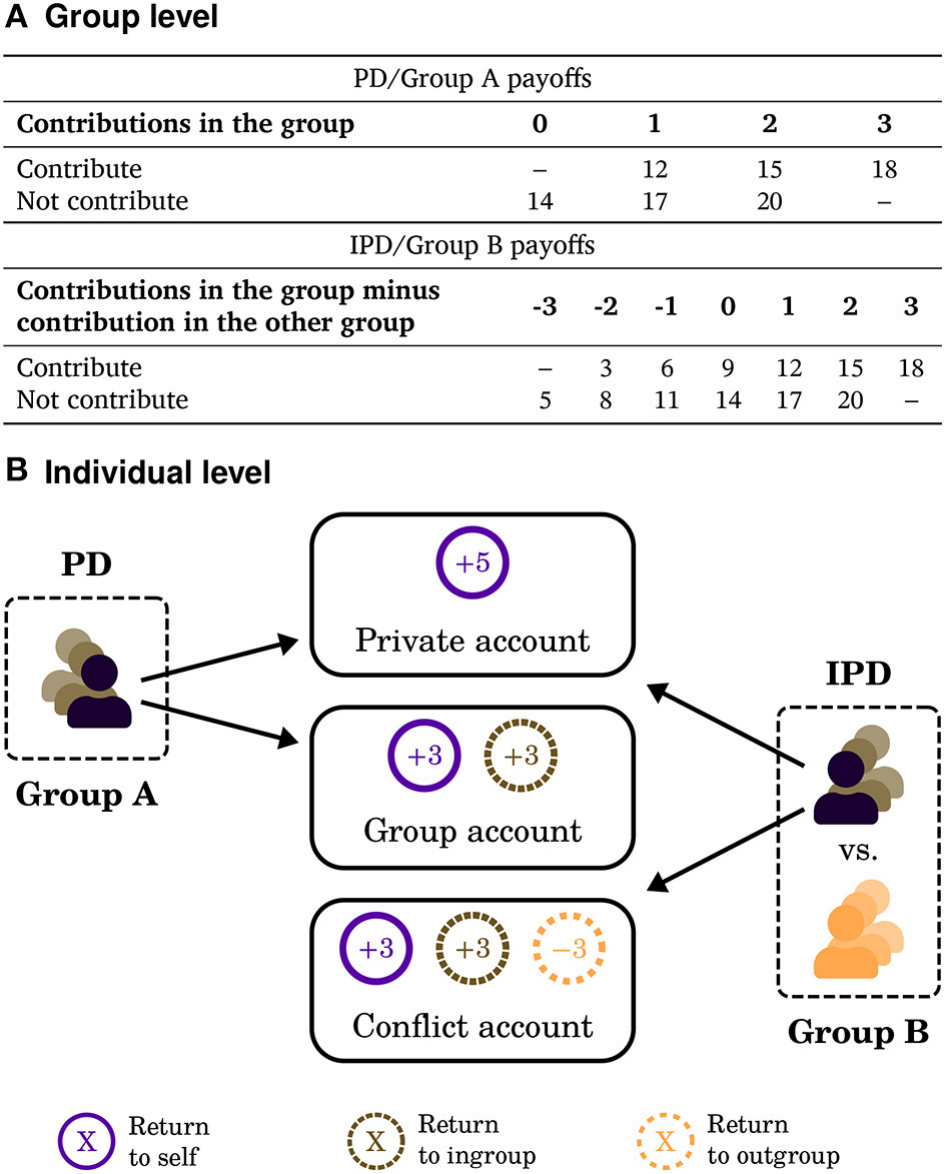
The IPD and PD games. Contributions are made to a group account in the PD and to a conflict account in the IPD. Panel **(A)** presents the payoff to the player based on the total contributions (columns) and her own contribution (rows). Panel **(B)** presents the same games as the change in payoff based on all players' choices between contributing and investing in a private account compared to a base endowment of 14. The player faces a choice between a private account and a group account. In the IPD, the choice is between a private account and a conflict account. The labels “Group A” and “Group B” refer to the asymmetric game that combines features of the PD and the IPD.

Experimental team games provide a laboratory model for studying intergroup conflict (Bornstein, 2003). Bornstein and Ben-Yossef (1994) provided experimental support for increased cooperation in conflict using the *Intergroup Prisoner's Dilemma* (IPD) game introduced by (Bornstein, 1992). As in the *Prisoner's dilemma* (PD) game, players can cooperate with the group by contributing to help their ingroup members. Cooperation, however, is modeled after mobilization in intergroup conflict in that it inflicts a loss on outgroup members. Figure 1 depicts the 3-player PD and the 6-player (two groups) IPD.

Because the intragroup structure is identical in the PD and the IPD, the comparison of cooperation levels in the two games provides a clean test for the effect of intergroup conflict on intragroup cooperation. Bornstein and Ben-Yossef (1994) found higher cooperation levels in the IPD (54.7%) compared to the PD (27.2%). This result has been replicated in several studies, and in some was moderated by personality type (Probst et al., 1999; Baron, 2001; Weisel and Zultan, 2016).

The studies that compared cooperation in the IPD and in the PD, such as Bornstein and Ben-Yossef (1994), framed the effects of contributions in the outgroup as a threat to the group as a whole. The typical instructions present the players' payoffs as a result of an explicit comparison of the *sum* of contributions in each of the two groups, hence the groups compete for a *common* group resource (see Figure 1A). Weisel and Zultan (2016) altered the instructions to shift the threat to the individual. A player's payoff was no longer based on a *comparison* of the total contributions made by the two groups, but instead increased with each contribution in the ingroup and decreased with each contribution in the outgroup (see Figure 1B). The actions of the outgroup now pose a threat to the individual, detached from the parallel effect on the other ingroup members. This variation in experimental instructions thus manipulates the perception of level of threat.

As in previous studies with the standard instructions, conflict increased cooperation. The effect was small overall, but strong and significant among pro-socials, with 55% contributions in the IPD compared to 33% in the PD. In the individual level condition, cooperation levels *decreased* with conflict from 61 to 31%. Note that the manipulation alters not only perception of conflict as collective threat, but also as collective *action* in the competition for resources with the outgroup. The experimental design addressed this issue by considering an asymmetric game, in which contributions in Group *B* harmed Group *A* members, but not vice versa (cf. Figure 1). Consequently, the manipulation affects the collective threat aspect in Group *A* and the collective action aspect in Group *B*. The manipulation in the instructions affected behavior in Group *A* as it did behavior in the symmetric IPD while having no significant effect on Group *B*, leading to the conclusion that the perception of collective threat—and not collective action—drives behavior (cf. De Dreu et al., 2016; De Dreu and Gross, 2019).

In the IPD game of Weisel and Zultan, (in press), participants allocated 10 tokens between private and conflict accounts. Similar to the binary version of the game, cooperation level increased from 38% under individual threat to 48% under group threat. Half of the groups communicated by electronic chat before making decisions. Independent judges rated whether cooperating in conflict, as reflected in the chats, was due to (a) competing with the outgroup; (b) defending the ingroup; (c) helping the ingroup; or (d) harming the outgroup.

We regressed cooperation levels (sum of tokens allocated to the conflict account by all group members) on the four reasons interacted with level of threat. In both conditions, discussions of helping the ingroup are strongly correlated with cooperation levels, whereas discussions of harming or competing with the outgroup do not correlate with cooperation. As could be expected, in the group threat condition, discussions of defending the ingroup are correlated with high cooperation levels. This pattern reverses under individual level threat. Here, groups who cooperated more tended to discuss defending the ingroup *less* than groups who cooperated less. The differing patterns between the two conditions clearly establish that changing the perceived level of threat fundamentally shifts the way in which people think of cooperation in conflict.

## 5. Discussion

Several observations suggest that a major factor in determining whether outside threat promotes or hinders ingroup cooperation is the extent of direct exposure to threat. More direct relation to casualties reverses the rally around the flag effect (Gartner, 2008b), and negative reactions to disaster are observed in proximity to inflicted harm (Tilcsik and Marquis, 2013). Berrebi and Yonah (2021) found that charitable contributions in the US generally increase in the aftermath of mass shootings, but decrease in the directly affected localities. Mass shootings are local secluded events not associated with concrete threat to people other than the immediate victims. Observed effects on philanthropic activity—which generally does not target victims of mass shootings—is attributable to psychological reactions rather than to responses to actual needs.

Theoretical accounts of reduced cooperation in face of threat often invoke *conservation of resources* theory, which states that “when confronted with stress [defined as the threat of a net loss of resources], individuals are predicted…to strive to minimize net loss of resources” (Hobfoll, 1989). We suggest that applying the distinction made in social identity theory between interpersonal and intergroup behavior to conservation of resources theory provides an underlying psychological mechanism for the seemingly opposing reactions to threat. Social identity theory posits that situational factors determine whether group affiliation—rather than personal perspective—governs the social behavior of individuals. In particular, intergroup conflict leads individuals to “behave toward each other as a function of their respective group memberships, rather than in terms of their individual characteristics or interindividual relationships” (Tajfel and Turner, 1979). When people construe conflict as a potential threat to their resources, they strive to maintain and protect their resources, reducing costly cooperation. When conflict is construed as an intergroup process, focus shifts to the threat to the group resources. Such perceptions of a common threat also strengthen group identity, leading people to view group and self resources as one, motivating them to maintain and protect group resources—increasing ingroup cooperation as a result.

According to our interpretation of the field studies discussed above, perceived level of threat varies with proximity—spatial, temporal, and social—to the victims. Proximity is naturally associated with myriad variables that guide cooperative behavior, making it difficult to establish a pure psychological effect of conflict on cooperation. Results from the laboratory experiments reviewed above provide clean evidence for such psychological effects. Framing conflict at the individual or group level manipulates perceived level of conflict while fixing the material effects of conflict. Thus, we can attribute any differences in behavior and its correlates between individual and group frames of conflict to the mediating effects of psychological perceptions.

## Author Contributions

OW and RZ contributed to all aspects of the research. All authors contributed to the article and approved the submitted version.

## Conflict of Interest

The authors declare that the research was conducted in the absence of any commercial or financial relationships that could be construed as a potential conflict of interest.
